# Nurses Coping with Stressful Situations—A Cross-Sectional Study

**DOI:** 10.3390/ijerph191710924

**Published:** 2022-09-01

**Authors:** Grażyna Iwanowicz-Palus, Mariola Mróz, Krystyna Kowalczuk, Beata Szlendak, Agnieszka Bień, Mateusz Cybulski

**Affiliations:** 1 Chair of Obstetrics Development, Faculty of Health Sciences, Medical University of Lublin, 4-6 Staszica St., 20-081 Lublin, Poland; 2 Department of Integrated Medical Care, Faculty of Health Sciences, Medical University of Bialystok, 15-096 Bialystok, Poland; 3 Center of Postgraduate Education for Nurses and Midwives, 02-106 Warsaw, Poland

**Keywords:** nurses, stress, coping styles, self-efficacy, psychological and physical well-being

## Abstract

Nursing belongs to the group of professions particularly exposed to stress. Since the ability to cope with stress is an important aspect of mental health, the aim of this study was to identify the types of nurses’ behaviours in terms of different coping styles used when dealing with work-related and psychosocial stress. The study was conducted among 1223 Polish nurses by means of a diagnostic survey, using the Coping in Stressful Situations Questionnaire (CISS), the Generalised Self-Efficacy Scale (GSES) and a questionnaire of the author’s own design. Three types of nurses were distinguished: Type 1 (non-harmonious/organised)—nurses with lower professional education, longer work experience, at least average severity of stress related to working conditions, the lowest GSES scores, and worse psychophysical condition, who focused on their own emotional state when faced with stressful situations. Type 2 (harmonious)—nurses with higher education, the lowest intensity of work-related stresses, the highest GSES scores, positive self-reported psycho-physical condition, most often using the task-oriented coping style in stressful situations. Type 3 (non-harmonious/disorganised)—nurses with shorter length of service in the profession, the highest intensity of work-related stress, average GSES scores, and poorer self-reported psycho-physical condition. The presented results may provide a basis for preventive measures to minimise stress and increase competence in coping strategies, thus contributing to improved psychological and physical well-being of nurses.

## 1. Introduction

In the 21st century, stress has become a natural, unavoidable part of our daily lives [1,2]. Stressors are varied and can be seen as negative events that inhibit the undertaking of new tasks or the continuation of ongoing tasks, but they can also be a kind of motivator to continue actions in the chosen direction [2,3]. Professional work is one of the most important human activities, as it satisfies needs, provides a sense of self-worth and is the basic source of income for employees and their families. It can be a source of life satisfaction, but also of dissatisfaction and stress [4,5,6,7].

Nurses are a vital link in the health care system. However, nursing is one of more demanding professions involving exposure to a number of stressors associated with the responsibility for the health and life of another human being [8,9,10,11]. Nursing requires constant focusing of attention, rapid responses, decision-making and the ability to perform many activities simultaneously, often under time pressure [4,12,13]. This is accompanied by the lack of promotion prospects, shift work and work overload, as well as an inadequate organisational structure, including staff shortages and low pay [6,14,15,16]. Other sources of stress include contact with patients’ families, working in a multidisciplinary team and within one’s own age-diverse professional group, as well as insufficient social support at work. [4,6,17,18]. The role of stressors also increases with the ever-increasing demands made by employers and recipients of medical services [4].

Stress coping is a constantly changing behavioural and cognitive effort directed at specific internal and/or external demands appraised as taxing or beyond one’s resources [19]. Endler and Parker (1990) distinguished three styles of coping with stress: task-oriented style (TOS—after the initial assessment, there is a tendency to make an effort to solve the problem); emotion-oriented style (EOS—undertaking action to reduce the emotional tension accompanying a stressful situation through wishful thinking, fantasising); avoidance-oriented style (AOS—rejecting thoughts about the fundamental problem, not allowing oneself to experience it and engage in solving the stressful situation: this style is expressed in two subscales, i.e., ESA—engaging in substitute activities—and SSC—searching for social contacts) [20,21,22,23].

Self-efficacy is also an important element in dealing with stress [24,25,26]. This factor enables proper assessment of the situation and the search for an effective coping strategy to deal with the encountered difficulties and obstacles. Self-efficacy can contribute to motivation to act and help achieve the individual’s intended goals [27]. High self-efficacy may reduce the tendency to show symptoms of work-related stress and increase competence to constructively deal with stress [28]. In addition, self-efficacy is positively correlated with organisation, commitment and job satisfaction, as well as better coping with difficulties [24,29].

Research on stress coping styles may be found in the literature. It shows, among other things, that nurses using the task-oriented style better deal with stressful situations. However, studies that would distinguish different types of nurses, depending not only on their stress-coping strategies, but also on the frequency of psychosocial and occupational stressors and personal resources, including self-efficacy, are missing [18,30,31].

The present study was designed considering the aforementioned stressors and potential difficult situations nurses are exposed to in their workplace, as well as the lack of scientific reports identifying types of nurses’ behaviours when faced with stress. In order to minimise stress, it has become essential to understand and categorise nurses’ behaviours in difficult situations, thereby providing a wider perspective for designing preventive measures.

The aim of this study was to distinguish the types of nurses’ behaviours in terms of their varied coping styles, work-related variables and their socio-demographic characteristics, as well as organisational, occupational and psychosocial stressors.

## 2. Materials and Methods

### 2.1. Study Design and Participants

This cross-sectional study was conducted in accordance with the guidelines for The strengthening the Reporting of Observational Studies in Epidemiology (STROBE). It was conducted in 2018 among 1223 nurses employed in medical institutions in Poland. Probability sampling was used. Research data was collected in randomly selected institutions offering postgraduate training for nurses across the country. The respondents were informed of the anonymity and voluntary nature of their participation in the diagnostic survey and that the results obtained would be used for research purposes only.

The respondents were informed about the course and purpose of the survey and instructed on how to complete the questionnaire. Those who agreed to participate in the study were given the survey questionnaire together with an informed consent form. The consent forms and survey questionnaires were left in special boxes which were opened after the survey was completed.

The inclusion criteria were as follows: a valid nursing licence and active professional practice. The exclusion criteria included lack of active nursing practice and incorrectly filled or incomplete questionnaire.

**Figure 1:**
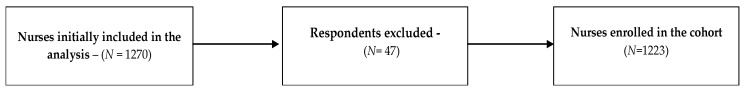
Flowchart of the recruitment process.

The group attending postgraduate training in the year of the study included 9845 nurses [32]. The minimum number of respondents was estimated at 370 (with a maximum error of 5% and a confidence level of 95%). A total of 1270 forms were distributed among the participants. A total of 1223 correctly completed survey questionnaires qualified for further statistical analysis (47 questionnaires were incompletely or incorrectly completed). The success rate of the data obtained was 96.29% (Figure 1).

### 2.2. Assessments

The study was conducted by diagnostic survey method. The research tool was a questionnaire consisting of two sections:

The Coping in Stressful Situations Questionnaire (CISS) consisting of 48 statements relating to various behaviours in difficult and stressful situations, divided into three scales representing stress-coping styles used by the respondents:

*Task-oriented style* (TOS) scale—identifies a coping style that involves planning and undertaking tasks to bring about a solution to a difficult situation.

*Emotion-oriented style* (EOS) scale—focusing on one’s own experiences, emotions.

*Avoidance-oriented style* (AOS)—identifies coping with stress that involves avoidance of thinking, experiencing emotions and difficult, stressful situations. This style can take two forms: engaging in substitute activities (ESA) or searching for social contacts (SSC).

Each scale consists of 16 questionnaire items and respondents can score between 16 and 80 points in each scale. The respondents answer the questions on a 5-point scale (1—never, 5—very often). Scores are converted to sten norms and are interpreted as follows: stens 1–4—low scores, stens 5–6—medium scores, stens 7–10—high scores. Reliability as measured by the α-Cronbach coefficient for the tool is 0.74–0.88. The α-Cronbach coefficient for the study group for individual scales was as follows: TOS—0.90; EOS—0.87; AOS—0.89; ESA subscale—0.86; SSC subscale—0.87 [23].

Generalised Self-Efficacy Scale (GSES)—a questionnaire comprising 10 questions with the possibility to choose one of four answers (1—no, 2—rather no, 3—rather yes, 4—yes). The scale measures the level of an individual’s overall belief in their effectiveness in coping with obstacles and difficult situations. The sum of all scores yields an overall self-efficacy index. Scores are interpreted based on sten norms: a sten score of 1–4 is defined as low and a sten score of 7–10 as high. The Cronbach’s α coefficient was 0.85 for GSES and 0.89 for the study group [33].

The author’s original questionnaire (Supplementary File) included questions to characterise the respondents and to assess their working conditions and work-related burdens. The respondents gave their opinion on a five-point Likert scale as to the frequency of certain work-related situations (1 for never and 5 for very often). The questions were grouped into 3 sections.

Stress related to the organisation of work: excessive responsibilities; rush; lack of time; lack of breaks; monotony of work; poor conditions; working extra hours; large number of patients under care; having to comply with inconsistent orders; poor conditions and insufficient organisation in the workplace; limited career opportunities; autonomy and independence in decision-making; safety in the workplace; stability of employment. Score range: min. 13, max. 65.

Psychosocial stress: providing the highest level of care to patients; receiving work instructions that are not in line with professional qualifications; lack of kindness, support and trust within the team; interpersonal conflicts; conflicts with supervisors, patients and their families; aggression from patients; respect from patients; recognition from supervisors. Score range: min. 12, max. 60.

Stress related to individual’s characteristics: high responsibility for health and life; frequent exposure to suffering/death; fear of losing work; lack of motivation to work; need to improve professional qualifications. Score range: min. 5, max. 25.

All procedures performed in studies involving human participants were in accordance with the ethical standards of the institutional and/or national research committee (the Bioethics Committee of the Medical University of Lublin: KE-0254/128/2017) and with the 1964 Helsinki declaration and its later amendments or comparable ethical standards. Informed consent was obtained from all individual participants included in the study.

### 2.3. Statistical Analysis

Statistical analyses were performed using *Statistica* version 13.3 and IBM SPSS Statistics (StatSoft Polska Sp. z o.o., Cracow, Poland). Characteristics of the study group was based on descriptive statistics of the analysed variables, including: mean (M), standard deviation (SD), numbers (n) and percentage (%). The significance of differences between the means was determined by one-way analysis of variance (F—Fisher statistics) and Student’s parametric t-test. The Pearson’s chi-square test (X^2^) was also used, which made it possible to estimate whether the distribution observed in a given group depended on another variable. Agglomerative cluster analysis and k-means cluster analysis were performed to distinguish subtypes in the study group of nurses and midwives. The level of statistical significance was set at *p* < 0.05.

## 3. Results

### 3.1. Characteristics of the Surveyed Nurses

Table 1 shows the characteristics of study participants. The respondents were predominantly nurses in the age range of 41–50 years (42.19%) living in a provincial city (39.33%), married or in an informal relationship (76.70%), with a master’s degree (40.31%), with work experience in the profession of 21 to 25 years (20.44%), (Table 1).

### 3.2. Assessment of Occupational Stressors and Nurses’ Stress-Coping Styles

In the study group of nurses, the highest scores in stress-coping styles were obtained for TOS (M = 55.49 ± 7.84), while the lowest for SSC (M = 17.08 ± 3.09). The mean score of general self-efficacy in the study group was 29.84 ± 3.69. As for the nurses’ self-reported occurrence of specific stressors, the highest severity was reported for the organisational dimension of professional work (42.81 ± 5.84), while the lowest severity was reported for the dimension related to individual’s characteristics (18.60 ± 2.76)—Table 2.

### 3.3. Types of Nurses in Terms of Stress-Coping Strategies and Perceived Psychosocial and Occupational Stressors

We used an agglomerative analysis followed by cluster analysis to classify the respondents. The clusters identified in the group of nurses express three types of stress coping strategies.

Type 1 (*n* = 383; 31.32%) was characterised by:

- Average intensity of stress associated with the organisation of work;
- The highest intensity of stress associated with psychosocial working conditions;
- Average intensity of stress associated with an individual’s characteristics.
- In terms of stress coping styles:
- The task-oriented, avoidance-oriented coping styles and engaging in substitute activities and searching for social contacts were used least frequently when facing stress;
- The emotion-oriented coping style was used relatively frequently.

Since nurses functioning according to this type perceived their work as very mentally taxing and focused on their own emotional state when in a stressful situation, this type of coping with stress was termed the non-harmonious/organised type (Table 3).

Type 2 (*n* = 385; 31.48%) was characterised by:

- The lowest intensity of stress related to work organisation and psychosocial working conditions, and work-related stress;
- The lowest intensity of stress related to the individual’s characteristics;
- In terms of stress coping styles:
- The task-oriented coping style was used most frequently;
- The emotion-oriented style was used least frequently;
- Coping style focused on avoidance and engaging in substitute activities and seeking social contacts was used relatively frequently.

Since nurses functioning according to this type rated organisational and psychosocial factors low and used a task-oriented style in stressful situations, this type was defined as the harmonious type (Table 3).

Type 3 (*n* = 454, 37.12%) was characterised by:

- The highest intensity of stress related to work organisation;
- Average severity of stress related to psychosocial working conditions;
- The highest intensity of stress related to the individual’s characteristics.
- In terms of stress coping styles:
- The task-oriented stress coping style was used relatively frequently;
- The most commonly used coping styles were emotion-oriented, avoidance-oriented; engaging in substitute activities and searching for social contacts.

Since nurses in this category reported the highest intensity of stressors (organisational, psychosocial and individual) and used the emotion-oriented style, avoided problem solving, engaged in substitute activities or searched for social contacts, this type was termed non-harmonious/disorganised (Table 3).

The analysis of variance presented in Table 3 confirmed that the nurses constituting the three distinct clusters differed in their preferred stress-coping style and in their perception of stress related to work organisation, psychosocial working conditions and stress related to individual characteristics. The reported effect strengths are high with the exception of the differences in stress related to individual characteristics.

### 3.4. Analysis of Sociodemographic Variables among Nurses with Different Stress-Coping Styles

Table 4 presents the results of a comparative analysis between nurses using three types of stress-coping strategies in terms of socio-demographic characteristics and work-related variables.

Type 1—nurses functioning according to the non-harmonious/organised type of coping had lower professional education, longer work experience in the profession, the lowest general self-efficacy and rated their overall health and psychophysical condition as lower (Table 4).

Type 2—nurses functioning according to the harmonious type of coping had higher professional education, the highest general sense of self-efficacy and rated their health, physical and mental condition highest (Table 4).

Type 3—nurses functioning according to the non-harmonious/disorganised type had shorter professional experience, average general sense of self-efficacy and rated their general health and psycho-physical condition as lower (Table 4).

## 4. Discussion

The present study assessed and distinguished specific types of nurses in terms of coping with stress, taking into account the severity of work-related stress, psychosocial working conditions and factors related to individual’s characteristics, including self-efficacy.

Our analysis revealed differences in sociodemographic variables in the identified types that characterise nurses. Education and length of service of the respondents were associated with their use of specific styles of coping with stress. Nurses belonging to the harmonious type had a higher level of education. The higher the educational level of nurses, the more often they used the task-oriented stress coping style. Kotarba and Borowiak (2018) also confirmed this relationship, indicating that nurses with higher educational levels cope better with occupational stress by using the task-oriented style [18].

According to Żuralska et al. (2015), unmarried nurses were more likely to choose confrontation-based strategies rather than avoidance. In our study, marital status did not significantly differentiate between respondents regardless of whether they represented the non-harmonious/organised, harmonious or non-harmonious/disorganised type [34].

Work experience was a differentiating variable in the identified types of nurses, in terms of work-related stressors and stress-coping styles. Nurses belonging to the non-harmonious/disorganised type had shorter work experience in the profession, and most often used the emotion-oriented and avoidance-oriented styles of coping. Our findings are in line with the data obtained by Perek et al. (2007), showing that the shorter the seniority of nurses, the more often they used the emotion-oriented style [35]. On the other hand, a study conducted among doctors by Basińska and Dziewiątkowska (2012) showed an inverse relationship. Namely, those with longer seniority were more likely to use avoidance and resignation strategies in stressful situations at workplace [36].

Kotarba and Borowiak (2018) showed that the value of task-oriented style among the nurses surveyed varied depending on age. The data obtained in the present study did not correspond with these results [18].

The levels of stress related to work organisation and psychosocial working conditions, as well as individual characteristics were also differentiating factors for the nurses surveyed. Nurses who reported the highest intensity of stress related to work organisation, an average intensity of stress related to psychosocial working conditions, the highest intensity of stress related to individual characteristics in a stressful situation used the emotion-oriented style, avoided solving their problems and engaged in substitute activities or sought social contacts. Medium intensity of work organisation-related stress, the highest intensity of stress related to psychosocial working conditions and average intensity of stress related to individual characteristics led nurses to focus on their own emotional state. Nurses who showed the lowest intensity of work organisation-related stress, the lowest intensity of stress related to psychosocial working conditions and the lowest intensity of stress related to individual characteristics used the task-oriented stress coping style. The results obtained correlate with the findings of C. Jenaro, N. Flores and B. Arias (2007), who conducted their study among Spanish social workers, indicating that the task-oriented coping style is associated with high job satisfaction [30]. The main goal of an individual using the emotion-oriented style is the need to reduce the psychological tension accompanying a stressful situation through wishful thinking, which has little effect and often results in a further increase in negative emotions and depressed mood [23]. In contrast, the task-oriented style allows difficulties to be overcome and triggers a positive attitude. Individuals who score high on this scale are above all able to plan and implement a solution to a problem. The task-oriented style is therefore considered to be the most optimal form of functioning in difficult situations [31].

In terms of stress related to cooperation with a supervisor, nurses who reported the most frequent occurrence of situations such as conflicts and receiving work instructions incompatible with qualifications were relatively more likely to use the emotion-oriented style than the task-oriented strategy. The results of the study did not correspond with the data obtained by Kaźmierczak et al. (2019), who found no difference between the lack of support from superiors and the choice of stress-coping strategies [37].

The non-harmonious/organised type was characterised by the highest intensity of stress related to psychosocial working conditions, which included lack of kindness, support and trust within the team. Nurses representing this type were least likely to use the task-oriented style in contrast to respondents in Kaźmierczak et al. (2019), who chose active stress coping strategies [37].

The nurses in our research who were found to have the highest levels of stress in terms of collaboration within the therapeutic team were the least likely to use the avoidance-oriented style and to engage in substitute activities, which was also reflected in the research data obtained by Kaźmierczak et al. (2019), according to which the lack of collaboration between members of a therapeutic team was significantly more likely to lead nurses to attend to something else [37].

The process of coping with stress depends on several factors, including social support and self-efficacy. In our study, we found that nurses belonging to the non-harmonious/organised type were least likely to seek social contacts despite being characterised by the highest intensity of psychosocial stress and the use of EOS. Similar results were obtained by Kaźmierczak et al. (2019), who found that nursing staff did not seek support from others when faced with team conflicts [37].

Available research shows that more coping skills in stressful situations and more adaptive behaviour in relation to professional work are reported among nurses characterised by higher levels of general self-efficacy. Nurses with high levels of self-efficacy show a better ability to cope with specific situations and are more likely to perform their tasks [38,39]. Those with high self-efficacy are also more satisfied with their work organisation. In addition, Andruszkiewicz, A. et al. (2011) proved that self-efficacy protects this professional group from experiencing too much work-related stress [24].

Our study showed that the more often the nurses used the task-oriented and avoidance-oriented style, the higher their sense of self-efficacy was. In contrast, the more often they used the emotion-oriented coping style, engaged in substitute activities and sought social contacts, the lower their general sense of self-efficacy was. Konaszewski et al. (2021) also indicated a positive correlation between self-efficacy and TOS [40]. The use of the task-oriented style by the professional group of nurses may indicate that they make an effort to solve a problem when facing stress.

Stress is a factor that interferes with the body’s equilibrium, indirectly affecting human health; therefore, while distinguishing the types of nurses in terms of stressors, sociodemographic variables and stress coping styles, attention was also paid to the important factor of the psychophysical condition of the representatives of this professional group. Based on our study, it was found that nurses using the emotion-oriented stress coping style and reporting more frequent stress rated their general health and psychophysical condition lower. In contrast, nurses functioning according to the harmonious type, using the task-oriented style, and who rated organisational and psychosocial stress as low, had better self-reported mental and physical condition and health status. Kaźmierczak et al. (2019) showed that somatic symptoms experienced by nursing staff and psychological factors were also significantly related to the choice of stress coping strategies [37]. Additionally, Kowalczuk et al. (2021) indicated that nurses using active stress coping styles were more likely to seek rational and positive solutions to difficult situations, which predisposes to maintaining good health [21].

Workplace stressors and coping styles are key factors influencing the mental and physical health of nurses (including anxiety and depression) and other negative emotions. Low self-reported psychophysical health condition leads to an increased absenteeism at work, staff fluctuation, and thus increased operating costs of healthcare facilities.

Since the available studies on coping styles presented by many authors do not distinguish between the modes of nurses’ functioning upon exposure to stress, the results presented here may provide a basis for preventive measures to minimise stress and increase coping competence, thus contributing to improved psychophysical well-being and the healthcare system.

The results may become a predictor for creating and introducing training sessions to develop task-oriented coping styles and improve self-efficacy. Furthermore, the introduction of measures to counteract unfavourable workplace phenomena, as well as the implementation of interpersonal training aimed at developing the ability to deal with stress, may increase the employee’s effectiveness and job satisfaction, and thus improve the self-reported health among the nursing personnel. Our findings may be a key element in the choice of methods and tools to ensure rational protection of nurses, thus providing them with a sense of security in their work environment.

### Strengths and Limitations of the Study

The strength of our study is that it collected data from across the country on coping styles used by nurses to deal with stress. The results obtained, using cluster analysis, allowed us to identify different types of nurses’ behaviours upon exposure to stress and factors predisposing to the use of the task-oriented style, which is the most desirable strategy.

As for the limitations, it should be pointed out that this was a cross-sectional study, therefore causal relationships could not. Furthermore, as the nursing profession is mainly practised by women, men did not participate in the study [41]. However, it would be relevant and interesting to conduct a study also in the group of active male nurses.

## 5. Conclusions

Nurses use varied coping styles when faced with stress, and they also show different degrees of stress related to work organisation, psychosocial working conditions and individual characteristics. Three types of coping with stress by the nurses surveyed were identified:

The harmonious type was the most acceptable type in nurses’ work, bringing together individuals who had low ratings of organisational and psychosocial stress and were most likely to use the task-oriented stress-coping style. These individuals also had the highest level of education and the highest levels of self-efficacy and a sense of being successful at work.

The type of behaviour described as non-harmonious organised was used by nurses with lower education, longer work experience and low self-efficacy. They perceived the work as highly mentally taxing and used the emotion-oriented coping style in difficult, stressful situations.

The non-harmonious disorganised type brought together nurses who had the shortest length of service, the highest sense of work overload and work-related stress, and a medium level of self-efficacy. In a stressful situation, they used the emotion-oriented style, avoided solving the problem by engaging in substitute activities or focusing on seeking social contacts.

Determining the types of nurses’ behaviours upon exposure to stress can provide a basis for interventions aimed at improving coping with stress, thus minimising the negative consequences of occupational stress. In order to counteract the perception of work as stressful, competence development should be promoted through continuing professional education and participation in coaching, aimed at developing the ability to apply the task-oriented style of coping with stress and building self-efficacy.

## Figures and Tables

**Table 1:** Participants’ baseline characteristics.

Participants’ Characteristics		Nurses	
		(n)	%
Age	≤30	274	22.40
	31–40	247	20.19
	41–50	516	42.19
	≥51	186	15.21
Residence	Urban—province capital	481	39.33
	Other cities	436	35.65
	Rural	306	25.04
Relationship status	Single	285	23.30
	Marriage/informal relationship	938	76.70
Education	High school education	289	23.63
	Bachelor’s degree	441	36.06
	Master’s degree	493	40.31
Seniority	≤10 years	330	26.99
	11–20 years	244	19.95
	21–30 years	493	40.31
	≥31 years	156	12.75

(n)—number, %—percentage.

**Table 2:** Mean scores of nurses’ stress-coping styles and their organisational, individual and psychosocial working conditions.

Variables		M	SD
Stress coping styles	Task-oriented style (TOS)	55.49	7.84
	Emotion-oriented style (EOS)	43.11	9.31
	Avoidance-oriented style (AOS)	45.73	7.81
	Engaging in substitute activities (ESA)	20.11	5.12
	Searching for social contact (SSC)	17.08	3.09
GSES	General self-efficacy	29.84	3.69
Types of loads	Increased stress related to work organisation	42.81	5.84
	Increased stress related to psychosocial working conditions	36.23	6.44
	Increased stress related to the individual’s characteristics	18.60	2.76

M—mean, SD—standard deviation.

**Table 3:** Comparative analysis between nurses functioning according to the three types of stress-coping strategies in terms of coping styles and specific types of stress.

Variables			Type 1 (n = 383; 31.32%)		Type 2 (n = 385; 31.48%)		Type 3 (n = 454; 37.12%)		Statistical Results of Variance Analysis			
			M	SD	M	SD	M	SD	F	*p*	η2	Post-hoc
Stress-coping styles		TOS	49.09	6.86	60.16	6.23	56.93	6.14	305.58	0.001	0.33	b > a, b > c, c > a
		EOS	40.44	6.75	36.07	6.36	51.33	6.72	600.04	0.001	0.50	c > a, c > b, a > b
		AOS	40.39	5.87	44.38	6.33	51.38	6.59	330.03	0.001	0.35	c > a, c > b, b > a
		ESA	17.84	4.12	17.99	4.18	23.82	4.44	275.03	0.001	0.31	c > a, c > b
		SSC	14.85	2.74	18.07	2.69	18.11	2.67	189.77	0.001	0.24	a < b, a < c
Stress	Related to the organisation of work		43.97	4.87	39.74	6.49	44.46	4.93	90.50	0.001	0.13	a > b, c > b
	Related to psychosocial working conditions		38.38	5.67	32.10	6.17	37.93	5.56	144.07	0.001	0.19	a > b, c > b
	Related to individual’s characteristics		18.01	2.61	17.63	3.29	18.78	2.89	2.87	0.056	0.03	-

(Type 1)—non-harmonious-organised, (Type 2)—harmonious, (Type 3)—non-harmonious-disorganised; (TOS)—task-oriented style, (EOS)—emotion-oriented style, (AOS)—avoidance-oriented style, (ESA)—engaging in substitute activities, (SSC)—searching for social contacts; (M)—mean, (SD)—standard deviation, (Me)—median; (F)—coefficient of variance analysis, (*p*)—statistical significance, (η2)—measure of effect strength.

**Table 4:** Comparative analysis between nurses functioning according to the three types of stress-coping strategies in terms of selected socio-demographic characteristics, condition and GSES.

Variables	Type 1		Type 2		Type 3		Statistical Results of Variance Analysis			
	M	SD	M	SD	M	SD	F	*p*	η2	Post-hoc
Age	5.59	1.76	5.45	1.92	5.32	1.87	2.21	0.009	0.003	-
Residence	1.86	0.78	1.80	0.80	1.89	0.77	1.33	0.262	0.002	-
Education	2.07	0.79	2.27	0.76	2.17	0.78	6.12	0.002	0.09	b > a
Relationship status	1.77	0.42	1.78	0.41	1.74	0.43	0.27	0.460	0.001	-
Length of service	4.47	1.90	4.21	2.05	4.07	2.08	4.18	0.015	0.09	a > c
General self-efficacy	28.51	3.98	31.54	3.32	29.52	3.17	75.48	0.001	0.11	b > a, b > c, c > a
Self-reported health status	3.61	0.69	3.83	0.65	3.64	0.71	11.52	0.001	0.02	b > a, b > c
Self-reported psychological well-being	3.60	0.69	4.01	0.62	3.57	0.75	50.39	0.001	0.08	b > a, b > c
Self-reported physical fitness	3.56	0.68	3.79	0.68	3.60	0.70	11.90	0.001	0.02	b > a, b > c

(Type 1)—non-harmonious-organised, (Type 2)—harmonious, (Type 3)—non-harmonious-disorganised; (M)—mean, (SD)—standard deviation, (Me)—median; (F)—coefficient of variance analysis, (*p*)—statistical significance, (η2)—measure of effect strength.

## Data Availability

Data are available upon reasonable request.

## Supplementary Materials

The following supporting information can be downloaded at: https://www.mdpi.com/article/10.3390/ijerph191710924/s1.
