# Supplementary material for: Nurses Coping with Stressful Situations—A Cross-Sectional Study
Source: Int J Environ Res Public Health. 2022 Sep 1;19(17):10924. doi: 10.3390/ijerph191710924 (PMC9518392; doi:10.3390/ijerph191710924)
Supplement: Supplementary file 1 [file ijerph-19-10924-s001.zip › ijerph-1856653-supplementary.pdf]

SURVEY QUESTIONNAIRE

1. Demographic data. Please put an "X" in the appropriate box

**l. p.**	**Item**		**"X"**
1	**Age**	≤30	
		31-40	
		41-50	
		≥51	
2	**Residence**	Urban-province capital	
		Other cities	
		Rural	
3	**Relationship status**	Single	
		Marriage/informal relationship	
4	**Education**	High school education	
		Bachelor's degree	
		Master’s degree	
5	**Seniority**	≤10 years	
		11- 20 years	
		21 - 30 years	
		≥31 years	

2. Questions characterizing the participants in terms of organizational, individual and psychosocial working conditions. Please indicate the frequency of certain situations related to your work as a nurse (*5-very often; 4-often; 3-rarely; 2-rather rarely; 1-never*).

**Stress/ Types of loads**		**Questions of the survey**	**Rating**				
			**5**	**4**	**3**	**2**	**1**
**1.**	**Related to the organisation of work**	1. Excess work responsibilities					
		2. Rush at work, lack of time for basic tasks					
		3. The need to submit to inconsistent orders					
		4. Autonomy and independence in making decisions					
		5. Limited promotion opportunities					
		6. No breaks at work					
		7. Monotony of work					
		8. Overtime work					
		9. Poor conditions and organisation in the workplace					

		10. Safety at workplace					
		11. Employment stability					
		12. A large number of patients requiring care					
		13. Working conditions enabling professional training					
**2.**	**Related to psychosocial working conditions**	1. Providing patients with the highest level care					
		2. Receiving official orders inconsistent with professional qualifications					
		3. Lack of kindness in the therapeutic team / employee team					
		4. Interpersonal conflicts					
		5. Lack of support from older colleagues / co-workers					
		6. Conflicts with the supervisor					
		7. Recognition from the supervisor					
		8. Lack of mutual trust in the therapeutic team / employee team					
		9. Conflicts with patients					
		10. Conflicts with patients' family members					
		11. Aggression from patients					
		12. Respect from patients					
**3.**	**Related to individual's characteristics**	1. Fear of losing job					
		2. Lack of motivation to work					
		3. High responsibility for other's health and life					
		4. Frequent exposure to suffering and death					
		5. The need to improve professional qualifications					
